# The skinny on skin: The role of skin‐aware professionals in skin cancer surveillance

**DOI:** 10.1111/jocd.16536

**Published:** 2024-09-01

**Authors:** Kyra Diehl, Jacob Nelson, Olivia Haddadin, Elizabeth Stoos, Autumn Shafer, Amy Mason, Deb Girard, Theresa Malcolm, Alan C. Geller, Emile Latour, Elizabeth Bailey, Jade N. Young, Hannah Zhao, Jordan Gillespie, Hailey Pfeifer, Claudia Lee, Moira Shea, Mallory DeCampos‐Stairiker, Jake Smith, Alyssa Becker, Gina N. Bash, Vikram Sahni, Yichen Fan, Elena Paz Munoz, David Baron, Nadia Popovici, Victoria E. Orfaly, Wenelia Baghoomian, Emilie Foltz, Kristen Kahlen, Stephanie Savory, Heidi Jacobe, Sancy A. Leachman

**Affiliations:** ^1^ Oregon Health & Science University Department of Dermatology Portland Oregon USA; ^2^ Oregon Health & Science University School of Medicine Portland Oregon USA; ^3^ University of Oregon Eugene Oregon USA; ^4^ IMPACT Melanoma Concord Massachusetts USA; ^5^ Department of Social and Behavioral Sciences Harvard T.H. Chan School of Public Health Boston Massachusetts USA; ^6^ Biostatistics Shared Resource Knight Cancer Institute, Oregon Health & Science University Portland Oregon USA; ^7^ Department of Dermatology Stanford University School of Medicine Stanford California USA; ^8^ John A. Burns School of Medicine University of Hawaii Manoa Hawaii USA; ^9^ Washington State University Elson S. Floyd College of Medicine Spokane Washington USA; ^10^ Memo Communications Seattle Washington USA; ^11^ Department of Dermatology University of Texas Southwestern Medical Center Dallas Texas USA; ^12^ Oregon Health & Science University, Knight Cancer Institute Portland Oregon USA

**Keywords:** education, melanoma, skin cancer, skin‐aware professional, training

## Abstract

**Background:**

Licensed nonmedical, skin‐aware professionals (e.g., hairdressers, massage therapists, etc.) have the potential to identify skin cancer, but baseline knowledge may not be sufficient to accomplish this goal. Following educational intervention, self‐efficacy is one of the best surrogate metrics for behavior change. Curricula that increase knowledge and confidence levels can improve screening behaviors, but few have been tested for efficacy in this population

**Aims:**

We assessed whether an online curriculum could reliably improve skin screening knowledge, attitudes, and behaviors of nonmedical professionals

**Patients/Methods:**

Skin‐aware professionals were recruited through the Oregon Health Authority and IMPACT Melanoma TM. Participants completed a pre‐survey, online training module, post‐survey, and one‐year follow‐up survey. We evaluated participants' indicated levels of concern for suspicious and nonsuspicious lesions relative to “gold standard” physician ratings. We also assessed confidence and self‐reported behavior change regarding talking to clients about skin cancer and recommending they see a provider to evaluate suspicious lesions

**Results:**

The pre‐survey was completed by 9872 skin‐aware professionals; 5434 completed the post‐survey, and 162 completed the one‐year follow‐up survey. Participants showed a significant improvement in ability to indicate the correct level of concern for all lesion types in concordance with “gold standard” physician ratings (*p* < 0.001). Participants reported increased comfort levels in discussing health‐related topics with their clients posttraining

**Conclusions:**

Our training module effectively increased skin‐aware professionals' knowledge, confidence, and concern for malignant lesions. Skin‐aware professionals may serve as a valuable extension of the skin self‐exam, but additional studies are needed to evaluate the impact of these curricula long‐term, including potential downstream consequences

## 1 INTRODUCTION

### 1.1 Skin‐self examinations (SSE) can improve early melanoma detection

Melanoma accounts for the majority of skin cancer‐related deaths; however, there is no national consensus on skin cancer screening in the USA.^1^ The total body skin examination is the safest and easiest screening test for skin cancer, but it is not routinely performed by primary care providers as part of the physical examination.^2^, ^3^ The SSE is an established method for efficient skin cancer screening, and efficacy is enhanced with the aid of another individual.^4^, ^5^, ^6^ When performed correctly, SSEs hold the potential to reduce melanoma‐related mortality by 63%.^7^, ^8^ However, only around 25% of patients perform SSEs, highlighting the need for increased awareness of its importance.^8^, ^9^

Key determinants influencing the actual performance of SSEs among high‐risk individuals include confidence in the ability to conduct self‐examinations, positive attitudes towards SSEs, and level of skin cancer knowledge.^6^, ^10^ In addition to self‐examination by lay people, there is also the consideration of the role of licensed nonhealthcare professionals who regularly observe their clients' skin as part of their daily services, also known as and hereafter collectively referred to as skin‐aware professionals. Skin‐aware professionals comprise a vast number of industries, including but not limited to, aestheticians/estheticians, hair stylists, barbers, massage therapists, tattoo artists, nail technicians, athletic trainers/instructors, and electrologists. With proper training on skin cancer detection, skin‐aware professionals may help bridge the gap between healthcare providers and the general population, particularly for those who may not conduct regular or efficient SSEs and/or lack regular healthcare access.

### 1.2 Skin‐aware professionals can help skin cancer screening efforts

While skin‐aware professionals lack the qualifications to make formal diagnoses, they are in a unique position to aid their clients in detecting skin cancer, especially in areas that are less visible during self‐examinations, such as the scalp and back. As these professionals observe skin regularly, they may be familiar with normal skin differences across a variety of skin tones and have the potential to learn how to recognize abnormal skin lesions across a large population of individuals.^11^ Skin‐aware professionals have a special opportunity to look for signs of skin cancer in clients who may not be proactively seeking medical attention. This allows them to play a role in enhancing early melanoma detection, reducing the time between detection and treatment, and potentially increasing survival rates within at‐risk populations.

### 1.3 Skin‐aware professionals often lack skin cancer knowledge

Skin‐aware professionals may serve as a valuable extension to the SSE, as they have more frequent interactions with clients compared to healthcare providers. Skin‐aware professionals are often approached by clients with skin concerns and report the desire to share health‐related information with clients. However, most skin‐aware professionals lack the training to convey accurate information because they receive little‐to‐no formal skin cancer education,^12^, ^13^ yet many skin‐aware professionals have the desire to obtain additional dermatologic education.^14^, ^15^ Providing education to these professionals has the potential to enhance their capacity to assist healthcare providers in skin cancer detection.

Despite not having received structured skin cancer education, many skin‐aware professionals are already assuming roles as informal skin educators for their clients. In a study of hair stylists, 63% reported discussing sun exposure with their clients.^16^ Additionally, 83% of Finnish tattoo artists reported conducting skin inspections prior to tattooing and 68% reported having referred a customer to a healthcare provider in the past year for a suspicious skin lesion.^14^ The top reasons for referral hesitancy, as reported by nail technicians, include fear of providing inaccurate information, potential client loss or dissatisfaction, and not knowing where to refer clients.^13^ Providing skin cancer education to these professionals can empower them to recognize potential malignant lesions, enabling them to make informed recommendations and referrals.

Educational models that emphasize the cultivation of self‐efficacy, or the perception of one's own ability to effect successful behavioral changes, create a sense of confidence in learners, leading to more desirable outcomes compared to models that solely focus on knowledge changes.^17^ A previous study that provided melanoma identification training to massage therapists showed significant improvements in knowledge scores of the ABCDE's of melanoma after training and increases in self‐reported comfort with melanoma identification.^18^ Increasing skin‐aware professionals' confidence in their ability to detect skin cancer may result in increased screening vigilance and encouragement of their clients to perform regular SSEs.

### 1.4 There is a lack of studies assessing skin cancer curricula among skin‐aware professionals as a collective group

While previous curricula involving subsets of skin‐aware professionals demonstrated improvements in skin cancer knowledge, confidence, and screening behaviors, there remains a lack of studies evaluating the effectiveness of curricula that target lesion detection among these professionals.^12^, ^13^, ^14^, ^15^ There is also a lack of longitudinal studies that evaluate the knowledge retention and lasting changes in the behaviors and attitudes of participants who receive skin cancer education. Furthermore, studies have only evaluated subsets of certain industries of these professionals separately. Therefore, there is an absence of studies analyzing all industries of skin‐aware professionals as a collective group. Our program aims to assess these professionals' ability to triage their clients to providers appropriately (i.e., refer suspicious cases without over‐referring nonsuspicious cases). While this is different from diagnostic lesion evaluations performed by dermatologists, it may lead to vital information about the ability of skin‐aware professionals to augment their clients SSE, while reducing unnecessary referrals for benign lesions.

### 1.5 The war on melanoma (WOM)™ esthetician pilot study improved skin cancer knowledge and confidence

Our online curriculum for skin‐aware professionals, which was developed utilizing the IMPACT Melanoma™ *Skinny on Skin* (SoS) training, expands upon results from our pilot study that trialed this curriculum with a sample of estheticians.^19^ In this study, 355 estheticians completed the pretraining survey, online training module, and posttraining survey. Most participants (85%–98%) correctly identified the level of concern for two out of three “extremely concerning” lesions on both surveys. However, their ability to recognize a benign lesion remained largely the same with 34% of estheticians indicating the correct level of concern before and after training. Participants were more likely to recommend their client to a medical professional if they previously attended a course on skin cancer (*p* = 0.012) or had greater than 1 year of work experience (*p* < 0.001). After training, there was a 35% increase in the number of estheticians that reported feeling very comfortable suggesting their client see a doctor for a suspicious lesion (52% pretraining vs. 87% posttraining).^19^

### 1.6 Objective

As a facet of Oregon Health & Science University's (OHSU) WoM™, our objective was fourfold: (1) to create a training program to teach skin‐aware professionals to recognize skin cancer and concerning lesions within their scope of practice, (2) to measure baseline skin cancer knowledge, behaviors, and attitudes and determine the impact of the training program on lesion detection, (3) to determine the retention of knowledge and change in behaviors of these professionals one‐year after training, and (4) to establish the “Skin Crew” in Oregon, a group of skin‐aware professionals interested in aiding early detection of melanoma. We hypothesized that each profession would have a positive attitude towards lesion detection and that the training program would show lasting improvements in knowledge, attitudes, and screening behaviors.

## 2 METHODS

### 2.1 Start seeing melanoma™ public health campaign and skin crew outreach

The study was approved by the institutional review board at OHSU (STUDY00019372). Recruitment for our education campaign was disseminated via: (1) the Oregon Health Authority (OHA) email and postal mail distribution lists, (2) OHSU's Skin Crew social media and print marketing resources, and (3) Quest Diagnostics “Beauty Campaign” of LinkedIn followers (directed towards aestheticians). Education was also delivered through an online multimedia platform with implementation through in‐person visits to businesses, schools, and large‐group and licensing board presentations. Through partnership with the OHA, 207000 emails were sent out between January and November 2022. In 2022, a LinkedIn campaign that included Skin Crew recruitment language was conducted in collaboration with the Quest Diagnostics marketing network. An additional Skin Crew marketing video was distributed through the University of Washington.

As part of the Start Seeing Melanoma™ public health campaign, OHSU's Department of Dermatology worked with the University of Oregon's health promotions group and a marketing specialist to formulate and distribute advertisements on the website, Instagram, and Facebook, as well as print media. During the first week of the campaign, a total of 35 892 postcards and 28 314 emails were sent out. Emails were opened by 33.6% of the people they were sent to. The social media campaign included both still in‐feed ads and animated story ads and was marketed to both metropolitan and rural areas of Oregon. The ads read as: “Melanoma stands out. It's the only cancer you can spot and stop with your own eyes. By checking your own skin, you could save your own life. Check your skin. You could spot cancer. Learn more at StartSeeingMelanoma.com #startseeingmelanoma.” Social media advertisements were targeted to individuals with the following interests, field of study, or job title: Cosmetics, Tattoos, Beauty salons, Spas, Yoga, Hair products, Weight training or Bodybuilding, Hair/Cosmetology, Salon Manager, Body Piercer, Hair Salon Manager, Salon/Spa Manager, Tattoo and Body Artist, Certified Massage Therapist (CMT), Nail Artist, Licensed Massage Practitioner (LMP), Barber Stylist or Esthetician. Results from the various advertisements were primarily measured in impressions (the number of times an ad is displayed).

Traffic to the website was analyzed in Google Analytics enabling the distinction between diverse sources of web traffic, including social media, email links, and direct entries. Because QR codes used on any marketing materials would be registered as a direct entry, discerning which visitors could be attributable to the postcard QR code was accomplished by utilizing unique URLs (unique uniform resource locators) for each campaign medium. Specifically, the capitalization distinction in the URLs (/SkinCrew/ for postcards QR codes versus /skincrew/ for other links) provided a novel method to infer the origin of page visits. This particularity allowed for the inference that a substantial number of these visits could be attributed to the use of postcards or direct QR code scans, given the capitalization in the URL provided in these materials.

With a primary focus on reaching professionals in Oregon, extensive outreach was performed in collaboration with the OHA to contact the leadership of each accreditation body of potential skin‐aware professionals. Discussions regarding the project purpose and planned interventions were held with each accrediting board of the respective professions, including the Board of Cosmetology, Board of Certified Advanced Estheticians, Athletic Trainer Registration Board, and Board of Electrologists and Body Art Practitioners. Three rounds of mass emails were sent to every professional licensed by the OHA. For licensed groups requiring Continuing Education (CE) credits, the *SoS* course was approved by the OHA as a CE provider.

**FIGURE 1:**
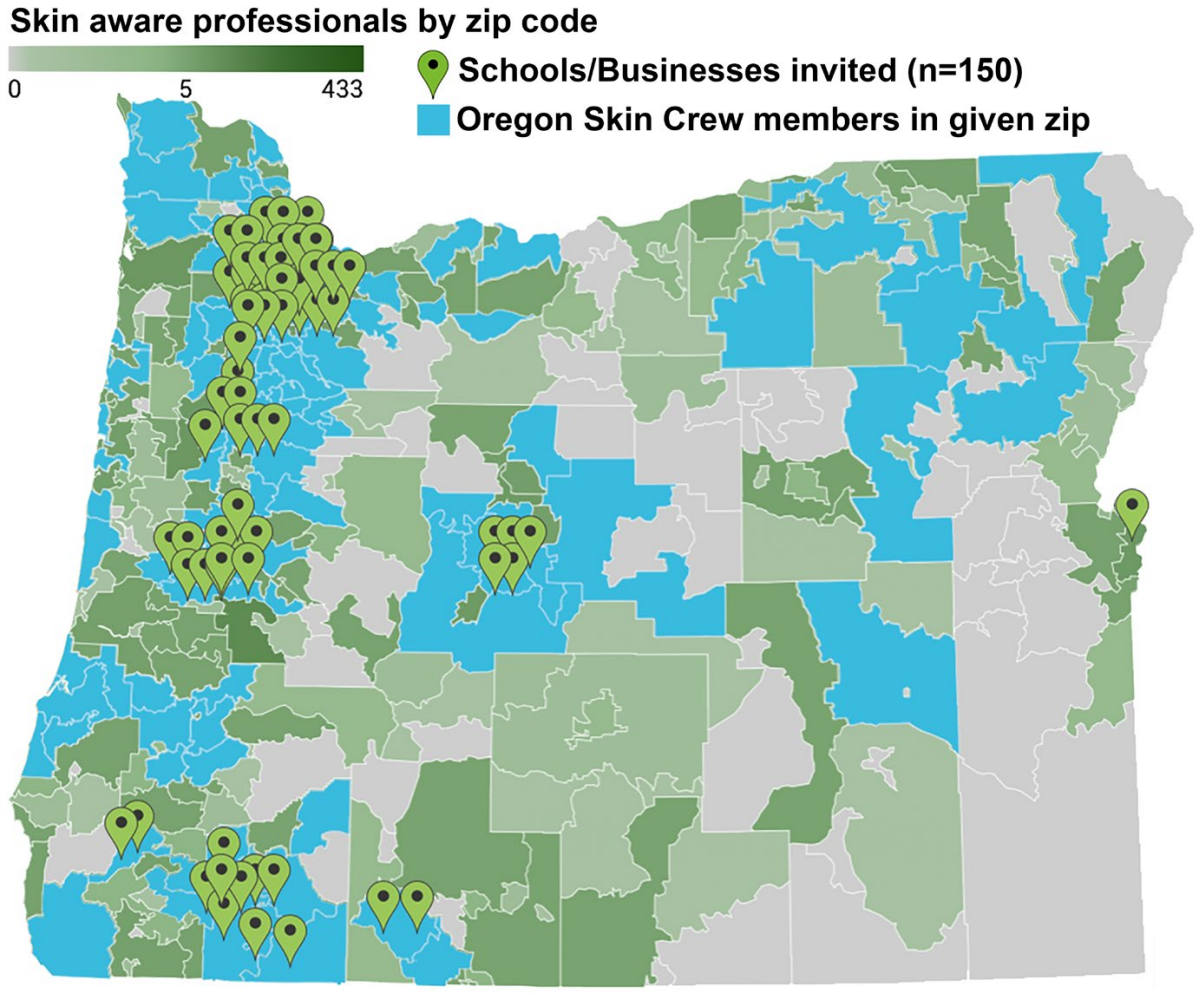
Skin crew outreach was conducted across oregon. Outreach to skin‐aware professionals was conducted in locations throughout Oregon with the green pins indicating locations where schools/businesses were invited to participate in the Skin Crew and the blue shaded areas indicating the zip codes where Oregon skin crew members are located.

To recruit more Skin Crew members, a comprehensive list of all 150 schools and businesses in the state were contacted: 104 (69%) cosmetology schools, 17 (11%) salons, 15 (10%) barber shop chains, 5 (3%) massage therapy schools, 4 (3%) tattoo artist schools, 1 (0.7%) hair styling business chain, 1 (0.7%) esthetician business chain, and 3 (2%) other related businesses. Locations in Oregon targeted through our outreach can be seen in Figure 1. A team of medical students, led by H.Z., additionally sent out over 4000 individualized emails to those who had not yet taken the *SoS* course to increase participation.

### 2.2 Components of the SoS education program

Participation in our curriculum was defined by whether an individual completed one or more components of the training, as described below. The *SoS* course is a 45‐minute online training program that was developed in collaboration with IMPACT Melanoma™, a nonprofit focused on melanoma early detection and prevention. The program consisted of three parts covering the identification of melanoma risk factors, recognizing the warning signs of melanoma, and effective ways to communicate with clients about melanoma. The curriculum involved visual recognition training with images of benign and malignant skin lesions, videos on the importance of early detection, and scenario‐based interactions on how to speak appropriately and within‐scope to clients. There were also pre‐curriculum, post‐curriculum, and follow‐up tests.

The pre‐ and post‐surveys were taken immediately before and after the training module, respectively. To assess for knowledge retention and screening behavior changes, a one‐year follow‐up survey was sent to participants who completed all three components: the pre‐survey, curriculum, and post‐survey. Participants who completed the follow‐up survey between February 2023 and August 2023 were included. As the majority of nonOregonian respondents were aesthetician/estheticians, an analysis was done comparing the demographics of Oregonian vs. nonOregonian aesthetician/esthetician respondents.

### 2.3 Pretraining, posttraining, and follow‐up survey design

**FIGURE 2:**
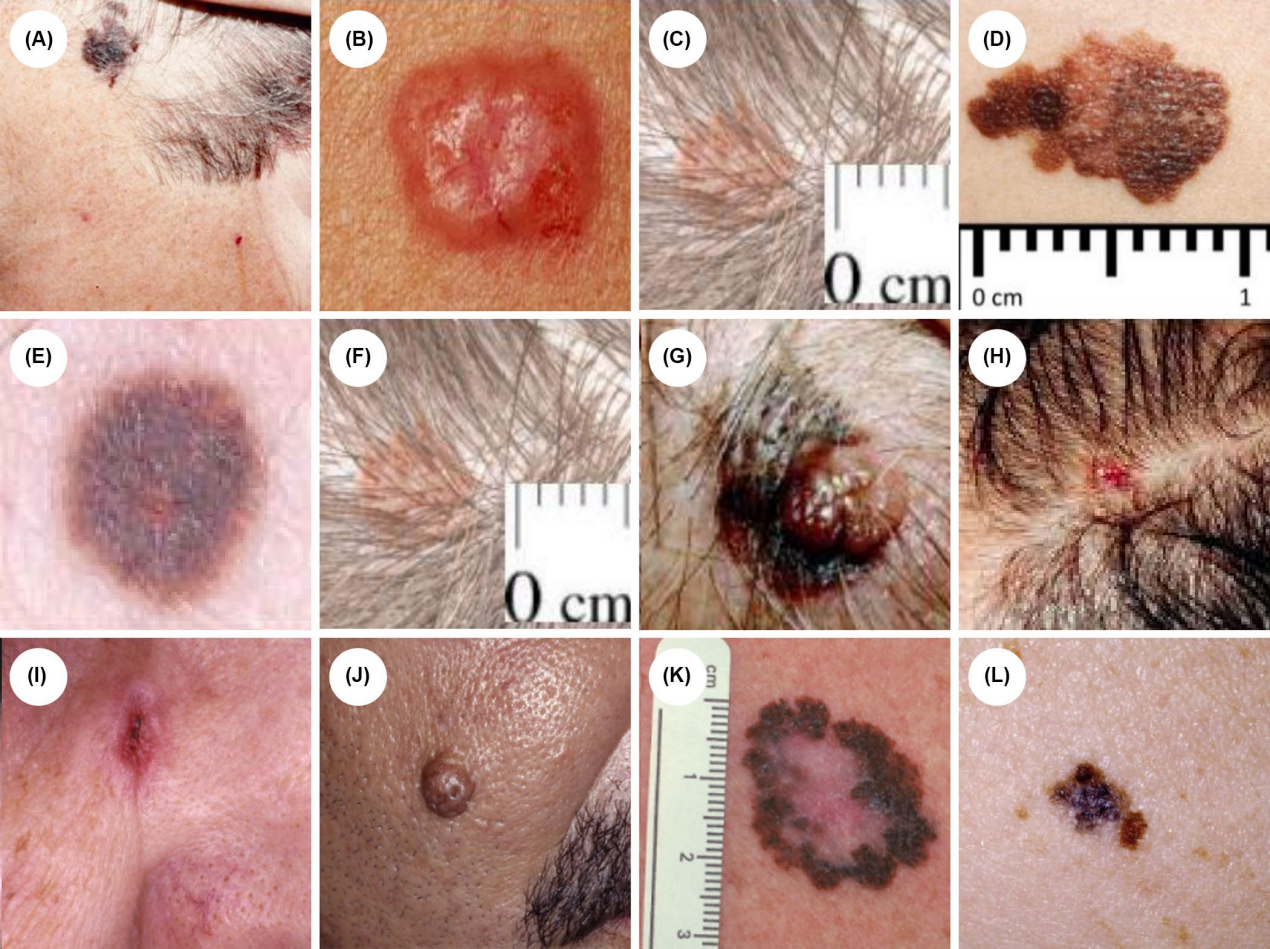
The Level of Concern for Concerning and Nonconcerning Lesions was Assessed. Images from pretraining (top row, A–D), posttraining (middle row, E–H), and follow‐up (bottom row, I–L) surveys. Each image included the prompt: “How concerned are you that this should be seen by a doctor or nurse?” Correct answers were as follows: (A) Extremely concerning (B) Extremely concerning (C) Slightly/Somewhat concerning (D) Extremely concerning (E) Extremely concerning (F) Slightly/Somewhat concerning (G) Extremely concerning (H) Extremely concerning (I) Extremely concerning (J) Slightly/Somewhat concerning K) Extremely concerning (L) Extremely concerning.

The complete set of questions on the pretraining, posttraining, and one‐year follow‐up surveys can be seen in Appendices A‐C. Participants were asked to assess their level of concern (extremely, slightly, somewhat, or not at all) for three sets of four pre‐selected lesions on the pretraining (Figure 2A–D), posttraining (Figure 2E–H), and follow‐up surveys (Figure 2I–L). Each test featured different lesion images, aside from the “slightly/somewhat concerning” image that was the same on the pre‐ and post‐surveys (Figure 2C,F). We aimed to maintain uniform difficulty across tests, with certain lesions being categorized as “more difficult” than others. There was complete consensus among the three expert dermatologists (SL, SS, and HJ) regarding levels of concern and level of difficulty. Respondents' levels of concern were compared to the expert consensus answers, which were considered a “gold standard” rating. The focus on visual identification of “concerning” lesions, rather than asking for a diagnosis of “melanoma” or “skin cancer” was intended to emphasize the need for these professionals to stay within their scope of practice (avoiding making a diagnosis). Participants did not receive correct answers after the tests to avoid test‐centric learning. Therefore, determining individual improvement of level of concern for specific lesions was not possible.

### 2.4 Survey data analysis

Descriptive statistics were used to summarize the survey responses for all respondents at each of the time points. Participants were matched by survey emails and results were analyzed for significance and data trends. Mixed effects models were used to examine the change in knowledge scores across time, and to adjust for specialty, student status, and industry experience. The analysis also used the program R for statistical computing. P‐values <0.05 were considered statistically significant. Statistical significance was also assessed using confidence intervals, where nonoverlapping intervals indicated significance.

## 3 RESULTS

### 3.1 Digital media and campaign recruitment of skin crew members

From January 2022 to December 2022, the social media campaign ads targeted towards skin‐aware professionals delivered 1 337 865 impressions via Facebook and 274 754 impressions via Instagram, for a total of 1 612 619 impressions. Over 9000 individuals have participated in the curriculum to date. Of these individuals, 1289 Oregonian members who have completed SoS training also consented to be listed as a Skin Crew member online. There were also 46 skin‐aware professional schools and related businesses that opted to join the Skin Crew (Figure 1). The Quest Diagnostics beauty campaign received 50 831 impressions and the University of Washington marketing video received 88 impressions.

**FIGURE 3:**
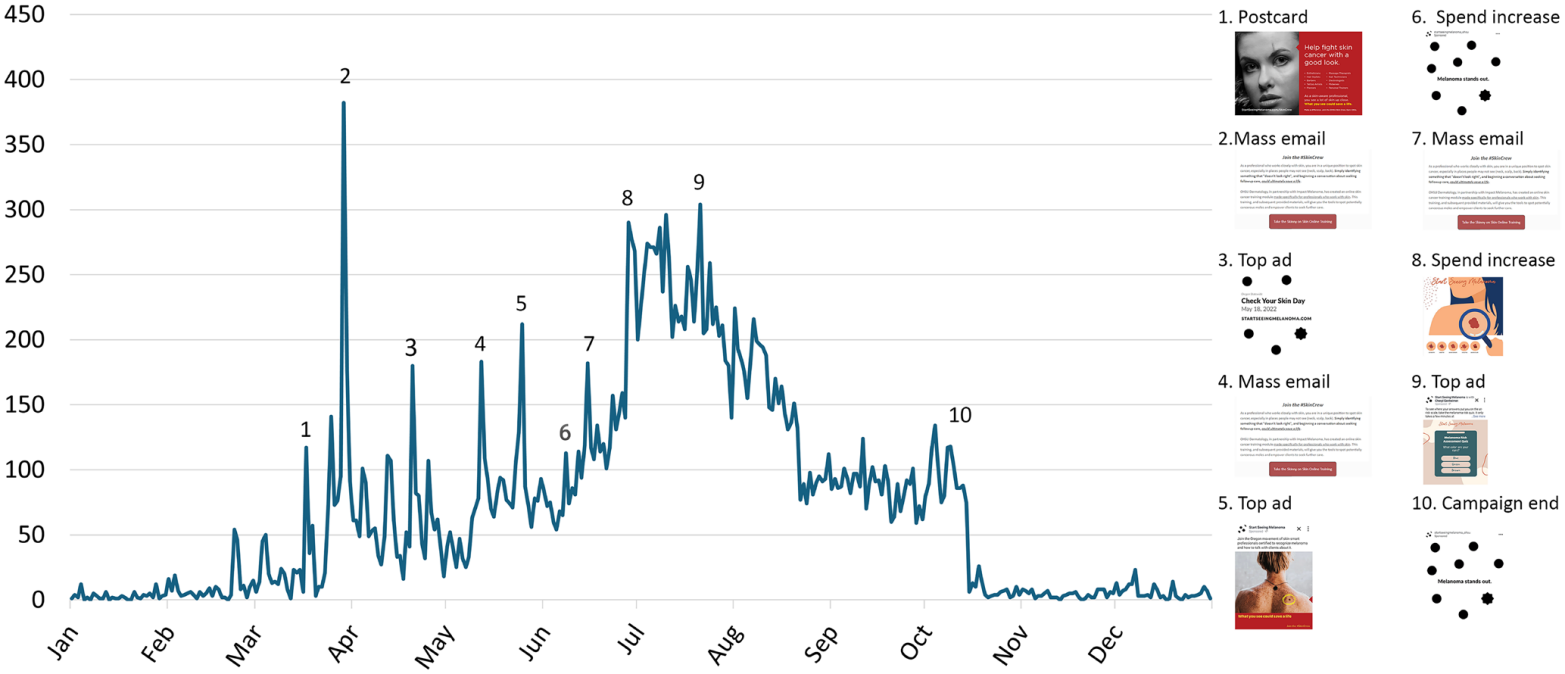
Social Media Ads Garnered the Majority of Website Visits. Daily Skin Crew page‐views on Startseeingmelanoma.com in 2022. This line graph represents the daily number of page‐views from January to December, annotated with key campaign events. Peaks in traffic coincide with specific promotional strategies: (1) Distribution of postcards, (2–4, 7) Sending of mass emails, (3, 5, 9) Deployment of top advertisements, (6, 8) Periods of increased advertising spend, (10) End of the campaign. The social media campaign occurred from March to October. Each numbered event corresponds to an increase in web traffic, indicative of the promotional impact on visitor engagement. The legend to the right show examples of promotional materials used at various stages, illustrating the diversity of approaches in the outreach campaign.

In 2022, outreach methods directing viewers to the Start Seeing Melanoma™ Skin Crew website resulted in a total of 27 390 website visits (Figure 3). The following sources were responsible for the website traffic: 66% (*n* = 17 949) from viewers of social media ads, 16% (*n* = 4481) from direct URL entries, 12% (3284) from email links, 3% (*n* = 895) from use of the QR codes on postcards, and 3% (*n* = 781) from Google searches.

### 3.2 National representativeness of skin‐aware professionals

Nationally, a total of 9782 skin‐aware professionals completed the pre‐survey, 5434 completed the post‐survey, and 162 completed the one‐year follow‐up survey, with 121 respondents matched across all time points (pre‐, post‐, and follow‐up surveys). Across all surveys, most respondents were female, White, and under the age of 50. Survey respondents were represented in each state, but residents of Oregon (13.2%) made up the largest portion of respondents, closely followed by South Carolina (13.1%), and Texas (10.4%), which were areas that received more marketing from IMPACT™. The complete demographic breakdown can be seen in Table 1.

**TABLE 1:** Survey respondent demographics and changes in screening behaviors and attitudes.

A. Survey respondent demographics						
Gender	Pretraining (*n* (%))		Posttraining (*n* (%))		Follow‐Up (*n* (%))	
Female	9231 (97)		5199 (97)		146 (90)	
Male	234 (3)		128 (2)		13 (8)	
Non‐binary	0 (0)		0 (0)		1 (1)	
Other	78 (1)		44 (1)		2 (1)	
Total Responses	9543 (100)		5434 (100)		162 (100)	
Age (years)	Pretraining (n (%))		Posttraining (n (%))		Follow‐Up (n (%))	
21 and younger	2489^25^		1397 (26)		4 (3)	
22–29	3426 (35)		1887 (35)		15 (9)	
30–39	1973^20^		1112^21^		38 (24)	
40–49	1006^3^		549 (10)		35 (22)	
50–59	553 (6)		322 (6)		29 (18)	
60–69	238 (2)		131 (2)		31 (19)	
70 and older	97 (1)		36 (1)		10 (6)	
Total Responses	9782 (100)		5434 (100)		162 (100)	
Race/ethnicity	Pretraining (*n* (%))		Posttraining (*n* (%))		Follow‐Up (*n* (%))	
Caucasian/White	4692 (49)		2816 (53)		129 (80)	
African American/Black	1994^21^		1030^19^		8 (5)	
Hispanic or Latino	2046^22^		1104^21^		13 (8)	
Asian or Pacific Islander	273 (3)		132 (3)		3 (2)	
Other	531 (6)		283 (5)		9 (6)	
Total Responses	9536 (100)		5434 (100)		162 (100)	
Years in the Industry	Pretraining (n (%))		Posttraining (n (%))		Follow‐Up (n (%))	
Less than 10	8513 (87)		4752 (87)		71 (44)	
10–20	674 (7)		379 (7)		36 (22)	
20 or more	595 (6)		303 (6)		55 (34)	
Total Responses	9782 (100)		5434 (100)		162 (100)	
Role in salon/business	Pretraining (n (%))		Posttraining (n (%))		Follow‐Up (n (%))	
Employee	2687 (28)		863 (16)		33 (20)	
Manager	197 (2)		90 (2)		9 (6)	
Owner	2156^22^		1083^20^		77 (48)	
Student	3738 (38)		2920 (54)		17 (11)	
Other	1004^3^		478 (9)		26 (16)	
Total Responses	9782 (100)		5434 (100)		162 (100)	
Professions	Pretraining (*n* (%))		Posttraining (*n* (%))		Follow‐Up (*n* (%))	
	All Respondents	OR Respondents	All Respondents	OR Respondents	All Respondents	OR Respondents
Aesthetician/Esthetician	6608 (68)	445 (35)	3776 (70)	283 (37)	56 (35)	18 (18)
Hair stylist	789 (8)	119 (9)	381 (7)	46 (6)	13 (8)	7 (7)
Massage therapist	463 (5)	357 (28)	324 (6)	259 (34)	49 (30)	46 (45)
Tattoo artist	114 (1)	97 (8)	76 (1)	67 (9)	8 (5)	8 (8)
Nail technician	99 (1)	22 (2)	55 (1)	13 (2)	3 (2)	2 (2)
Athletic trainer/Instructor	74 (0.8)	25 (2)	31 (1)	16 (2)	4 (3)	2 (2)
Electrologist	26 (0.3)	11 (1)	15 (0.3)	8 (1)	5 (3)	3 (3)
Barber	31 (0.3)	12 (1)	10 (0.2)	5 (1)	0 (0)	0 (0)
Other	1578^9^	189 (15)	766 (14)	72 (9)	24 (15)	17 (17)
Total Responses	9782 (100)	1277 (100)	5434 (100)	769 (100)	162 (100)	103 (100)
B. Oregon vs. non‐Oregon Aesthetician/Estheticians						
Gender	Non‐Oregon (*n* (%))		Oregon (*n* (%))			
Female	5913 (96)		425 (96)			
Male	66 (1)		11 (2)			
Other/Not answered	183 (3)		9 (2)			
Age	Non‐Oregon (n (%))		Oregon (n (%))			
21 and younger	1595 (26)		69 (16)			
22 to 29	2565 (42)		114 (26)			
30 to 39	1292^21^		102 (23)			
40 to 49	497 (8)		82 (18)			
50 to 59	167 (3)		53 (12)			
60 to 69	25 (0.4)		22 (5)			
70 and older	21 (0.3)		3 (1)			
Race/Ethnicity	Non‐Oregon (n (%))		Oregon (n (%))			
Non‐Hispanic White	2519 (41)		301 (68)			
Non‐Hispanic Black	1550^25^		10 (2)			
Hispanic	1376^22^		64 (14)			
Non‐Hispanic Asian and Pacific Islander	206 (3)		26 (6)			
Non‐Hispanic American Indian and Alaska Native	63 (1)		11 (2)			
Other/Prefer not to answer	448 (7)		33 (8)			
Years in the Industry	Non‐Oregon (*n* (%))		Oregon (*n* (%))			
Less than 10	5833 (95)		343 (77)			
10–20	226 (4)		59 (13)			
20 or more	103 (2)		43 (10)			
Role in salon/business	Non‐Oregon (n (%))		Oregon (n (%))			
Employee	1799 (29)		137 (31)			
Owner	1226^20^		153 (34)			
Manager	83 (1)		10 (2)			
Other	3054 (50)		145 (33)			
Total	6162 (100)		445 (100)			
C. Anatomic Locations of Customers Skin Being Screened for Skin Cancer by Respondents Pretraining						
Location	<25%	25–50%	51–75%	>75%	Total Responses	
Face	2124 (31)	778 (12)	737 (11)	3144	6783 (100)	
Scalp	3209 (55)	1012^10^	652 (11)	973 (17)	5846 (100)	
Neck	2321 (35)	1053^9^	1072^9^	2124 (32)	6570 (100)	
Back	2829 (48)	964 (17)	833 (14)	1231^21^	5857 (100)	
Legs	3148 (56)	929 (17)	633 (11)	907 (16)	5617 (100)	
Hands	2846 (47)	1102^11^	834 (14)	1309^22^	6091 (100)	
Feet	3504 (66)	701 (13)	405 (8)	708 (13)	5318 (100)	
Stomach	3676 (73)	583 (12)	317 (6)	434 (9)	5010 (100)	
D. Comfort With Sharing Health‐related Knowledge						
Comfort level	Pretraining (*n* (%))		Posttraining (*n* (%))		Follow‐Up (*n* (%))	
Quite/Extremely	5053 (53)		5342 (98)		123 (76)	
Somewhat/A little	3607 (38)		70 (1)		59 (36)	
Not at all	888 (9)		22 (0.4)		3 (2)	
Total Responses	9548 (100)		5434 (100)		162 (100)	
E. Pretraining Screening Behaviors						
Behavior					Pretraining (n (%))	
Number of customers that have asked respondent to look at a spot on their skin			None		6231 (66)	
			1–4		2636 (28)	
			5–8		459 (5)	
			9 or more		222 (2)	
			Total responses		9548 (100)	
Number of times respondents recommended a customer see a doctor or nurse			None		6354 (67)	
			1–4		2713 (28)	
			5–8		330 (4)	
			9 or more		151 (2)	
			Total responses		9548 (100)	
Percentage of customers respondent talked to about skin cancer			Less than 25%		7386 (77)	
			25–50%		1064^4^	
			51–75%		515 (5)	
			Over 75%		583 (6)	
			Total responses		9548 (100)	
Frequency that respondent discusses health‐related topics with customers			Never		1840^19^	
			Rarely/Sometimes		3788 (40)	
			Often/Always		3920 (41)	
			Total responses		9548 (100)	
Whether respondent currently sees customers in a salon/business			Yes		4730 (72)	
			No		1809 (28)	
			Total responses		6539 (100)	
Whether respondent has previously attended a skin cancer education course			Yes		3170 (35)	
			No		5889 (65)	
			Total responses		9059 (100)	
F. One‐Year Follow‐Up Screening Behaviors						
Behavior					Follow‐up (*n* (%))	
Received additional skin cancer education since *Skinny on Skin*						
Yes					13 (8)	
No					142 (92)	
Total Responses					155 (100)	
Number of clients that asked for respondents to look at a spot on their skin						
None					24 (15)	
1 to 2					24 (15)	
3 to 5					28 (18)	
6 to 10					26 (16)	
11 to 20					12 (8)	
More than 20					46 (29)	
Total Responses					160 (100)	
Number of clients that respondents recommended to see a healthcare provider for a skin lesion						
None					43 (27)	
1 to 2					50 (31)	
3 to 5					31 (19)	
6 to 10					18 (11)	
11 to 20					10 (6)	
More than 20					8 (5)	
Total Responses					160 (100)	
Percentage of clients who were referred to see a healthcare provider that reported a biopsy that showed skin cancer						
None					106 (66)	
1 to 2					32 (20)	
3 to 5					13 (8)	
6 to 10					6 (4)	
11 to 20					0 (0)	
More than 20					3 (2)	
Total Responses					160 (100)	
Frequency that respondents perform self‐skin exams						
Daily					13 (8)	
Weekly					23 (15)	
At least once a month					45 (29)	
At least every 3 months					23 (15)	
At least every 6 months					14 (9)	
At least once a year					27 (17)	
Total Responses					155 (100)	
Frequency that respondent discusses skin cancer with customers						
Never					1 (1)	
Rarely/Sometimes					88 (57)	
Often/Always					66 (43)	
Total responses					155 (100)	
G. Follow‐Up Self‐Reported Skin Cancer Diagnoses Detected After Referral						
Number of reported diagnoses	Malignant melanoma (*n* (%))	Basal cell carcinoma (*n* (%))	Squamous cell carcinoma (*n* (%))	Pre‐cancer (*n* (%))	Unknown Type (*n* (%))	
0	44 (62)	44 (65)	44 (71)	41 (59)	45 (74)	
1	20 (28)	12 (18)	10 (16)	11 (16)	10 (16)	
2 or more	7 (10)	12 (18)	8 (13)	17 (25)	6 (10)	
Total Responses	71 (100)	68 (100)	62 (100)	69 (100)	61 (100)	

Oregonian respondents represented a broad range of skin‐aware industries, while nonOregonian respondents were predominantly aestheticians/estheticians, reflecting our nationwide marketing emphasis towards beauty schools. An analysis comparing the Oregonian and nonOregonian aestheticians/esthetician respondents revealed demographic differences that prevent direct group comparison. Specifically, a larger portion of the nonOregonian aesthetician/estheticians were <30 years old and had <1 year of industry experience. These demographic differences in the nonOregonian cohort can be attributed to the substantial proportion of beauty school respondents.

### 3.3 The SoS curriculum improved baseline skin cancer knowledge

#### 3.3.1 Baseline knowledge improved post‐curriculum

**FIGURE 4:**
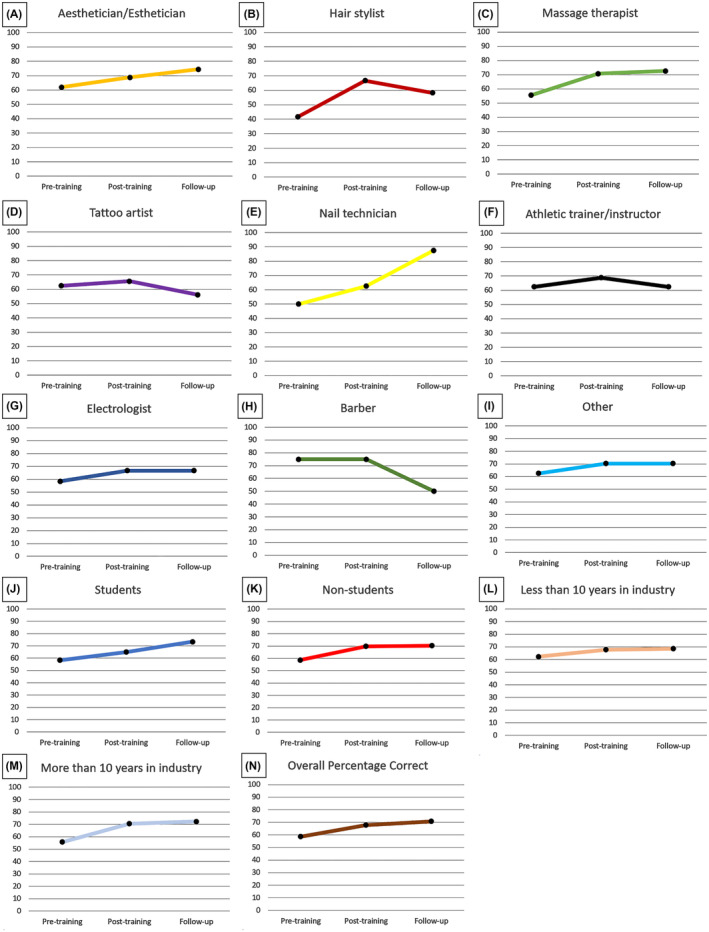
Most skin‐aware industries improved their baseline knowledge of skin cancer after training and retained it 1 year later. Changes in test scores were measured in survey respondents who were matched across all three surveys (*n* = 121) and compared across each specialty: Aesthetician/estheticians (*n* = 40) (A), hair stylists (*n* = 6) (B), massage therapists (*n* = 41) (C), tattoo artists (n = 6) (D), nail technicians (*n* = 2) (E), athletic trainer/instructors (*n* = 4) (F), electrologists (*n* = 3) (G), barbers (*n* = 1) (H), and other specialties (*n* = 16) (I). Changes in test scores were also compared in respondents who were students (*n* = 15) (J), non‐students (*n* = 106) (K), had less than 10 years of experience in the industry (*n* = 55) (L), and had more than 10 years of experience in the industry (*n* = 66) (M). Overall changes in test scores across all participants (*n* = 121) can be seen in panel (N). All specialties showed an increase in test scores from pretraining to posttraining except for the barber. Most specialties retained higher knowledge at follow‐up, except barbers, tattoo artists, hair stylists, and athletic trainer/instructors. The overall percentage correct significantly increased from pretraining (58.7%) to posttraining (69.2%) to follow‐up (70.7%) (*p* < 0.001). Respondents with more than 10 years in the industry had a significant improvement in test scores across all three surveys (*p* = 0.047). The change in percentage correct across all three surveys was not significant for students vs. nonstudents (*p* = 0.533).

**FIGURE 5:**
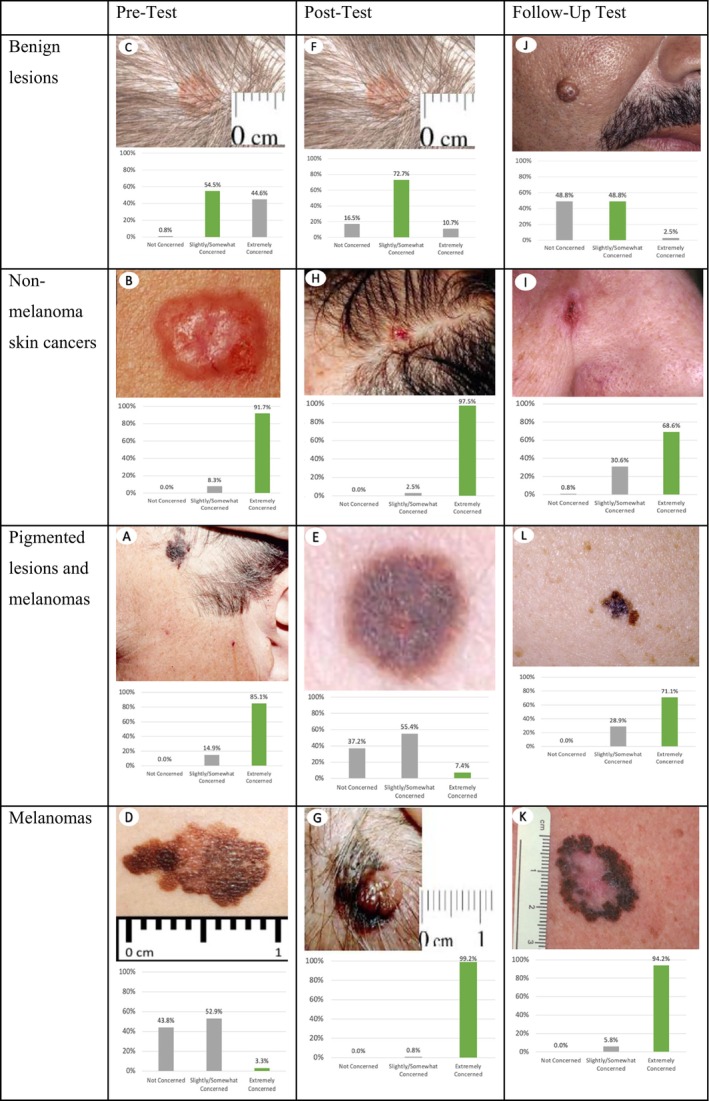
Respondents Reported an Accurate Level of Concern for Most Skin Cancers. Pre‐test, post‐test, and follow‐up test performance on each image‐based question shown in Figure 2 were measured in survey respondents who were matched across all three surveys (n = 121) and matched based on the following lesion severities: Benign lesions (C, F, J), nonmelanoma skin cancers (B, H, I), pigmented lesions and melanomas (A, E, L), and melanomas (D, G, K). The bar highlighted in green represents the correct answer choice and expert's level of concern for each lesion.

Knowledge changes were assessed in professionals who responded to all three surveys (*n* = 121). Test score improvements were significant across all three surveys for matched respondents (*p* < 0.001). From pre‐ to post‐test specifically (*n* = 5434), test scores significantly increased by 10.5% (Pre‐test: 58.7%; 95% CI: 55.5% to 61.8%; Post‐test: 69.2%; 95% CI: 67.0% to 71.4%) (Figure 4N). Most participants indicated the correct level of concern (“extremely concerned”) for two out of three “extremely concerning” lesions on the pre‐test (85.1%, 91.7%) (Figure 5A,B, respectively) and post‐test (99.2%, 97.5%) (Figure 5G,H, respectively). The third “extremely concerning” lesion that yielded lower scores was categorized as the most difficult of the three “extremely concerning” lesions. Pretraining, only 3.3% of participants were able to correctly identify the appropriate level of concern for the more difficult “extremely concerning” lesion (Figure 5D) and only 7.4% correctly identified the level of concern for a separate difficult lesion posttraining (Figure 5E). As for the “slightly/somewhat concerning” lesion, 54.5% correctly identified the appropriate level of concern pretraining (Figure 5C), and 72.7% identified the correct level of concern posttraining (Figure 5F).

#### 3.3.2 Knowledge at one‐year follow‐up remained improved from baseline

One‐year later, follow‐up respondents answered 70.7% of questions correctly (95% CI: 66.6%–74.7%). This was a significant increase from pre‐test scores (58.7%; 95% CI: 55.5%–61.8%), as well as an increase from post‐test scores (69.2%), although the latter was not statistically significant (95% CI: 67.0%–71.4%) (Figure 4N). Follow‐up respondents identified the correct level of concern “extremely concerning” for all three types of malignant lesions: nonmelanoma skin cancers (68.6%), pigmented lesions and melanomas (71.1%), and melanomas (94.2%) (Figure 5I,L,K, respectively) and 48.8% correctly identified the level of concern “slightly/somewhat concerning” for the benign lesion (Figure 5J).

#### 3.3.3 Skin cancer knowledge varied by profession

Aestheticians/estheticians comprised the majority of respondents in both the pretraining (67.6%; *n* = 6608) and posttraining (69.5%; *n* = 3776) surveys. At follow‐up, they remained the largest group at 34.6% (*n* = 56), closely followed by massage therapists at 30.2% (*n* = 49). For Oregonian respondents specifically, aestheticians/estheticians made up the largest percentage in the pretraining (34.8%; *n* = 445) and posttraining (36.8%; *n* = 283) surveys, but massage therapists led follow‐up survey completion (44.7%; *n* = 18). Across all three surveys, barbers and electrologists made up the smallest percentage of respondents, both overall and among Oregonians (Table 1).

In our study, the same curriculum was given to individuals across nine skin‐aware industries and knowledge changes were evaluated using a uniform method. When pre‐ and post‐survey respondents were matched, significant score improvements were observed for each occupation (*p* < 0.001). However, this significance was not observed within occupations when respondents were matched from post‐test to follow‐up (*p* = 0.301), or across all three surveys (*p* = 0.478). To evaluate knowledge changes by industry, the percentage of correct level of concern responses was stratified by occupation and matched by respondents of all three surveys (*n* = 121) (Figure 4A–I). Nail technicians had the largest improvement in scores, with an increase of 37.5% (50.0% pretraining, 62.5% posttraining, 87.5% follow‐up) (Figure 4E), followed by massage therapists with an increase of 17.1% (55.5% pretraining, 70.7% posttraining, 72.6% follow‐up) (Figure 4C). Barbers demonstrated the most substantial decrease in scores of 25.0% (75.0% pretraining, 75.0% posttraining, 50% follow‐up) (Figure 4H), followed by tattoo artists with a decrease of 6.3% (62.5% pretraining, 65.5% posttraining, 56.2% follow‐up) (Figure 4F).

#### 3.3.4 Skin cancer knowledge varied by student status

Our skin‐aware cohort included a diverse range of industry roles (employees, managers, owners, students, other). Students constituted the largest percentage of respondents in both the pre (38.2%; *n* = 3738) and post‐surveys (53.7%; *n* = 2920). Business owners were the largest percentage of follow‐up respondents (47.5%; *n* = 77) (Table 1).

We assessed for significant differences in knowledge change between matched student and nonstudent respondents by survey. Both students and nonstudents demonstrated significant score increases from pre‐ to posttraining (*p* = 0.004); however, there was no significant score change from posttraining to follow‐up (*p* = 0.270), or for respondents of all three surveys (*p* = 0.533). The percentage of correct level of concern was stratified by student status and matched by respondents of all three surveys (*n* = 121) (Figure 4J, K). Test scores of nonstudent respondents significantly increased by 11.1% from pre‐ (58.7%; 95% CI: 55.3–62.1) to post‐test (69.8%; 95% CI: 67.5%–72.2%) (Figure 4K). Although not statistically significant, student respondents also had a score increase of 6.7% from pre‐ (58.3%; 95% CI: 50.5%–66.1%) to post‐test (65.0%, 95% CI: 58.6%–71.4%) (Figure 4J).

#### 3.3.5 Skin cancer knowledge varied by years of experience in the industry

As our cohort included individuals with many industry roles, including students, we further evaluated responses based on years of experience. We categorized those with less than 10 years of experience as “early‐career” respondents and those with more than 10 years of experience as “experienced” respondents. “Experienced” professionals made up the largest percentage of respondents in the pre‐ (87.1%; *n* = 8513) and post‐surveys (87.3%; *n* = 4752); however, “early‐career” respondents were the largest percentage at follow‐up (56.2%; *n* = 91) (Table 1).

We assessed for differences in knowledge change by years of experience in matched respondents from each survey. Both “early‐career” and “experienced” respondents demonstrated significant score improvements across all three surveys (*p* = 0.047), as well as specifically from pre‐ to posttraining (*p* < 0.001), but not from posttraining to follow‐up (*p* = 0.835). The percentage of correct level of concern was stratified by years in industry and matched by respondents of all three surveys (*n* = 121) (Figure 4L,M). “Early‐career” respondents had a 6.3% score increase from pre‐test (62.3%; 95% CI: 58.3%–66.3%) to follow‐up test (68.6%; 95% CI: 62.6%–74.6%) (Figure 4L). “Experienced” respondents demonstrated a greater and statistically significant score improvement of 11.6% from pre‐test (55.7%; 95% CI: 51.1%–60.3%) to follow‐up test (72.3%; 95% CI: 66.8%–77.9%) (Figure 4K).

### 3.4 Skin‐aware professionals' screening behaviors and attitudes improved after participating in the SOS curriculum

**FIGURE 6:**
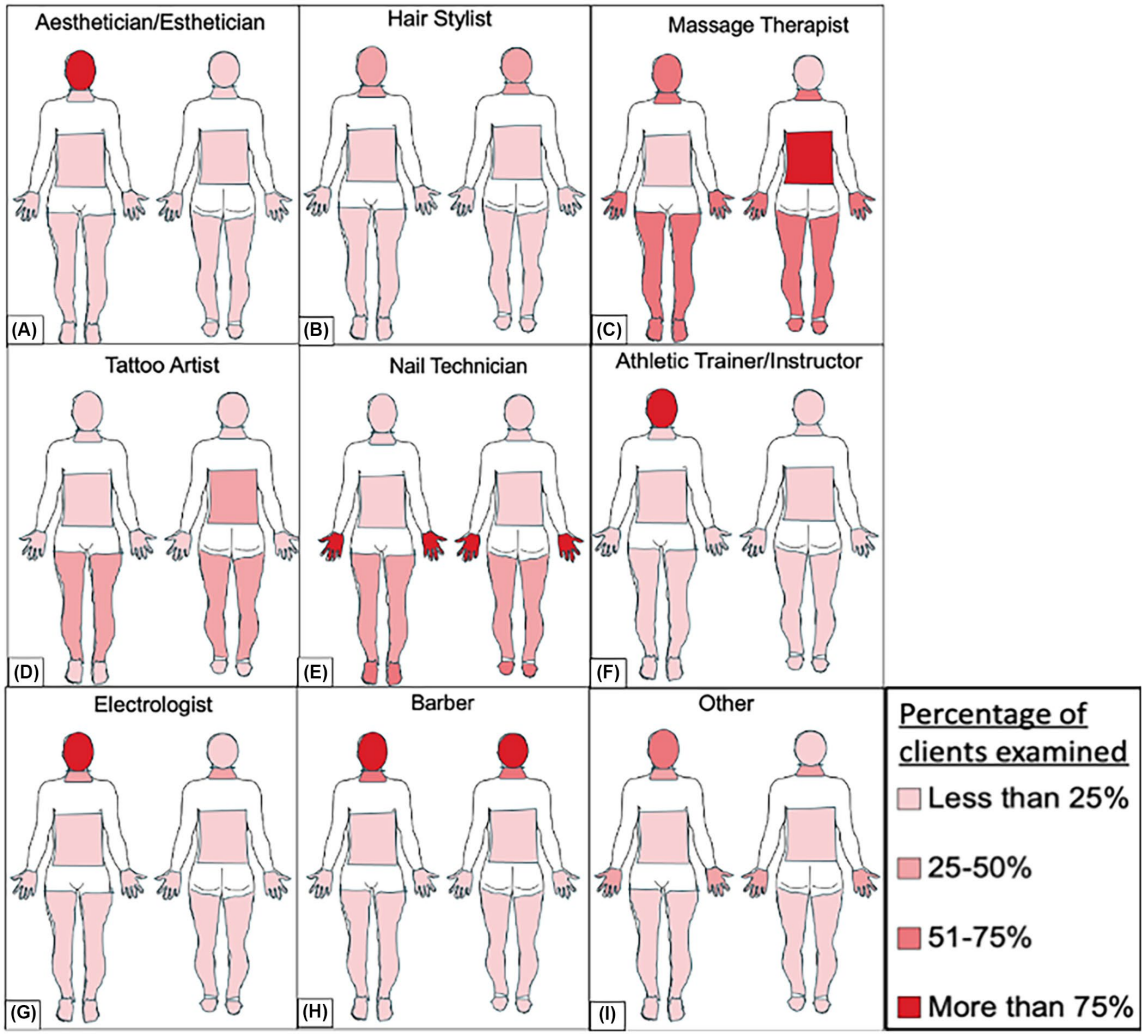
At Baseline, Most Skin‐Aware Professionals Are Only Evaluating the Face or Body Parts Specific to Their Profession for Skin Cancer. Pre‐survey respondents (*n* = 9782) were asked the percentage of their clients (less than 25%, 25%–50%, 51%–75%, and more than 75%) they examined skin in for the following locations: Scalp, face, neck, stomach, back, hands, legs, and feet. The client percentage response that received the majority (over 50%) of answers is shown for each body location for the following specialties: Aesthetician/estheticians (A), hair stylists (B), massage therapists (C), tattoo artists (D), nail technicians (E), athletic trainer/instructors (F), electrologists (G), barber (H), and other specialties (I). For example, the majority (over 50%) of nail technicians reported examining the nails in greater than 75% of their clients. Massage therapists are the only professionals that examine a substantial number of their clients in areas other than the face and head. Several specialties (aesthetician/estheticians, athletic trainer/instructors, electrologists, and barbers) examine the face in greater than 75% of their clients.

At baseline, participants were asked what percentage of customers they screened for suspicious skin lesions in an array of different body locations (face, scalp, neck, back, legs, hands, feet, and stomach). Out of those locations, the face was the only area that the majority of skin‐aware professionals (57.3%) reported screening for suspicious lesions in over 50% of their clients. Furthermore, a majority of respondents (>50%) reported screening less than 25% of their clients for skin lesions on the scalp, legs, feet, and/or stomach**.** This was further stratified by profession (Figure 6), with most professions reporting them either only screen customers' faces and/or the body parts that correlate most with services they provide. For example, over 50% of nail technicians reported screening the hands of over 75% of customers (Figure 6E). Overall, massage therapists reported examining the largest percentage of their customers' skin in more body areas compared to any other profession (Figure 6C).

Following participation in the curriculum, skin‐aware professionals exhibited improved comfort in sharing knowledge with their clients. However, the frequency that they reported having these discussions remained relatively unchanged. Out of 9548 respondents on the pretraining survey, 52.9% (*n* = 5053) reported being “quite/extremely” comfortable sharing health‐related knowledge with their customers and 41.0% (*n* = 3920) reported “often” or “always” discussing health‐related topics with them. At one‐year follow‐up, 75.9% of respondents (123/162) reported they were “quite/extremely” comfortable sharing knowledge with their clients and 42.5% (66/155) reported “often” or “always” discussing skin cancer with them (Table 1).

At follow‐up, 93.4% of respondents reported performing SSEs at least once a year or more often. Since completing training, 85.0% of respondents screened one or more clients for skin cancer and 73.1% referred one or more clients to a provider. Fifty‐four respondents (33.8%) reported that at least one of the clients they had referred for evaluation had received a skin cancer diagnosis. Regarding the type of diagnosis that clients reported receiving (pathology reports were not requested), 38.1% reported a malignant melanoma diagnosis, 35.2% reported a basal cell carcinoma diagnosis, 29.0% of respondents reported a squamous cell carcinoma diagnosis, 40.5% reported pre‐skin cancer diagnoses, and 26.2% reported skin cancer diagnoses of unknown type (Table 1).

## 4 DISCUSSION

### 4.1 A combination of outreach methods successfully recruited skin crew members

OHSU's WoM™, in collaboration with IMPACT Melanoma™, implemented an outreach program and educational curriculum to a broad spectrum of skin‐aware professionals. To increase reach and efficacy, our campaign used outreach methods both online and in‐person, including emails, postcards, school/business visits, social media, and our Skin Crew website. Of those, social media garnered the most traffic to our website and therefore was the most effective method both in reaching individuals and creating action. A systematic review of the use of social media in recruitment for research studies found that when compared to traditional methods, social media is the best recruitment method for hard‐to‐reach populations and observational studies. However, the effectiveness of social media for recruitment of study participants was highly variable and dependent on demographics of the study population.^20^ Further research is needed to determine which method of recruitment is the most effective in terms of efficacy, cost, and population reach.

### 4.2 Improving the screening abilities of skin‐aware professionals yields benefits to both the healthcare system and the general population

In Oregon, there are approximately 50 000 licensed, skin‐aware professionals and only 235 dermatologists (1:200 ratio).^21^ This highlights the potential impact that effective skin cancer training can have on the general population. By aiding in early detection and reducing unnecessary healthcare visits, this education has the potential to lessen the strain on healthcare resources. Furthermore, with the rising shortage of dermatologists and worsening access issues,^22^, ^23^ improving the screening abilities of skin‐aware professionals and promoting regular SSE performance can help ease this burden on both system‐ and population‐wide levels. Although lacking the diagnostic expertise of dermatologists, through learned pattern recognition, these professionals can recognize signs concerning for malignancy. The use of nonhealthcare professionals to aid in recognizing health conditions is not unique to skin cancer. For example, teachers are often relied on to detect neurocognitive and mental health disorders in their students,^24^ and dentists are educated to screen for oral cancer during cleanings.^25^ However, professionals in both industries acknowledge the need for more training to feel competent in recognizing signs of these conditions.^24^, ^25^

### 4.3 Does skin cancer education lead to over‐screening?

Our curriculum aimed to harness the benefits of equipping skin‐aware professionals with an improved ability to recognize skin cancer, while avoiding the potential risks of creating over‐confidence and over‐screening. Whereas excessive screening could lead to a strain on the healthcare system with unnecessary referrals, the harm from missing a melanoma is much more significant for the affected individual. Conversely, a false positive screening outcome that results in an unnecessary referral doesn't necessarily cause true harm to that individual because the consulting provider would presumably identify that lesion as benign. At least in our dataset and using our curriculum, there was not an increase in skin‐aware professionals' assigning higher‐levels of concern for benign‐appearing lesions, nor lower‐levels of concern for malignant‐appearing lesions. This suggests that this curriculum may be attaining an appropriately improved level of confidence for referral of malignant lesions without leading to over‐screening.

### 4.4 Does skin cancer education create over‐confidence?

Participants in our curriculum successfully demonstrated sustained improvements in their ability to detect cancerous lesions, along with increased comfort in discussing health‐related topics with clients. However, skin cancer education inherently poses the risk of creating over‐confidence in the ability to distinguish between malignant and benign skin lesions. While our curriculum aims to improve confidence levels, excessive levels of confidence may translate to an increased amount of unnecessary referrals for benign lesions. However, in our study, only 11% of respondents at posttraining and 3% at follow‐up (Figure 5F, J, respectively) indicated “extreme concern” for a benign lesion. On the other hand, excessive confidence may also result in skin‐aware professionals reassuring clients for malignant lesions, leading to missed or delayed diagnoses. Our results do not indicate an increased chance of this outcome, as 0% of respondents indicated “no concern” for melanoma lesions both posttraining and at one‐year follow‐up (Figure 5G, K, respectively). This aligns with similar results from a melanoma education program involving nail technicians, which showed significant post‐education increases in the number of technicians referring clients for melanoma, with no change in the likeliness to refer benign lesions.^13^ This promisingly suggests that effective skin cancer education does not necessarily result in increased unnecessary referrals. Moving forward, it is crucial to exercise caution in preventing overconfidence through skin cancer education and directing participants to utilize methods such as tele‐health when contemplating referrals for a skin lesion, in order to avoid stepping outside their scope of practice.

### 4.5 Nail technicians and massage therapists improved the most in ability to recognize skin cancers

Our study assessed knowledge changes using a uniform method across nine skin‐aware industries. Each occupation had significant test score improvements from pre‐ to posttraining (*p* < 0.001). Nail technicians demonstrated the largest score improvement from pretraining to follow‐up (Figure 4E), followed by massage therapists (Figure 4C). Interestingly, massage therapists also reported examining their clients' skin in the most body locations compared to the other professions in our cohort (Figure 6). This may suggest that skin‐aware professionals who regularly observe larger areas of their clients' skin are more likely to improve their ability to recognize malignant lesions. Conversely, barbers had the largest score decrease from pretraining to follow‐up (Figure 4H) although, they also consistently represented the smallest percentage of respondents across each survey. This suggests a potential lack of interest in skin cancer education among the barbers in our cohort, which could explain their lack of improvement compared to others. More studies are needed to understand and determine any potential explanations for the variability in learning among these industries.

### 4.6 Knowledge improved the most in nonstudents and those with more work experience

Nonstudent respondents and respondents with more years of industry experience had more substantial improvements in posttraining scores compared to their counterparts. This suggests that they may be more receptive to this training, possibly because they were not exposed to skin cancer education during their initial education, and therefore had more room for improvement. Additional studies are needed to understand the skin cancer education that these professionals received in their prior education, as well as to identify methods for improvement in future curricula. This includes examining changes in curricula over time, assessing for variations in content, and understanding the role that these professionals are taught to play in skin cancer detection.

### 4.7 Skin cancer knowledge was largely retained one‐year after training

There is a lack of studies evaluating long‐term knowledge retention after receiving skin cancer education. To address this gap, we conducted a follow‐up survey to assess knowledge retention and behavior changes one‐year after training. Follow‐up test scores increased further from both the pre‐ and post‐tests. This suggests that the relatively small number of participants who completed the one‐year follow‐up survey (*n* = 162) continued to improve over time, perhaps due to an inherent interest in the screening process. Our curriculum may have stimulated these participants to pay more attention to their clients' skin, consequently improving their ability to identify concerning and nonconcerning lesions on the follow‐up test. This is further supported by the large percentage of follow‐up respondents (85%) who reported they screened clients for skin cancer since training. However, it is important to acknowledge that the follow‐up survey was only sent to participants who completed the pre‐survey, curriculum, and post‐survey in their entirety. Therefore, they were likely the more motivated and interested individuals compared to other respondents and the results possibly reflect this bias as highly motivated respondents are more likely to actively engage with the curriculum and apply the skills and knowledge they learned.

### 4.8 Limitations

One aspect of this study that must be considered involves the real‐world context in which it took place. Despite successfully receiving over 1 000 000 impressions and recruiting over 1200 skin crew members, the campaign was executed amidst the challenges of the COVID‐19 pandemic. This likely constrained our marketing team's ability to recruit a greater number of study participants, given the closure or financial strain experienced by numerous smaller, skin‐aware businesses due to the pandemic. In addition, this directly impeded our marketing team's efforts to recruit skin‐aware influencers, further limiting our campaign's outreach potential.

Although our participants demonstrated high accuracy in identifying the correct level of concern for two out of three “extremely concerning” lesions across all three surveys, fewer correctly identified more subtle lesions. Through post‐hoc analysis, we discovered that sub‐optimal image quality in the pre‐ and post‐tests may have been a confounding factor and limitation in the present study, particularly in the post‐test. More subtle attributes of the skin lesions may not have been readily apparent to nondermatologists, and difficulty in recognizing these concerning features could explain the lack of improvement from pre‐ to posttraining for certain respondents. The images in the follow‐up test were chosen to ensure improvement in the image quality, which should have led to an improvement in ability to indicate the correct level of concern. However, the ability to identify the correct level of concern for the benign lesion slightly decreased from 55% on the pretest (Figure 5C) to 49% at follow‐up (Figure 5J), suggesting that while image quality could have made some of the more subtle qualities of skin lesions less discernible on the pre‐test, the difference was not large enough to undermine the findings of this study.

## 5 CONCLUSION

A large proportion of the U.S. population are clients of skin‐aware professionals. Oftentimes these professionals see their clients on a more frequent basis and have a closer relationship than they do with their primary care providers or dermatologists. This puts them in a unique position regarding their ability to assist with skin cancer screening and detection of early‐stage malignant lesions. Studies have shown that these professionals are receptive to receiving skin cancer education and many already examine their clients' skin for suspicious lesions. However, few have the education required to recognize and differentiate malignant from benign skin lesions.^12^, ^13^, ^14^, ^15^, ^16^, ^18^ Furthermore, many lack the confidence to discuss health‐related topics with their clients or avoid referring their clients to physicians out of fear of being wrong.^13^ Our results demonstrate that ability of skin‐aware professionals to recognize malignant lesions and their confidence in discussing health‐related topics with clients is improved after skin cancer education training, and many of these professionals report checking their client's skin. This suggests that these curricula are beneficial in both improving knowledge and attitudes of skin‐aware professionals regarding their ability to screen for skin cancer. Providing this education to professionals across various skin‐aware industries has the potential to increase screening of at‐risk patients, allowing for earlier detection and better prognoses among the general population.

The skin cancer education curriculum validated by this study is available for free to any skin‐aware professional at: https://www.ohsu.edu/war‐on‐melanoma/skinny‐skin‐elearning

## AUTHOR CONTRIBUTIONS

The authors confirm that all authors have significantly contributed to the manuscript herein as defined on the journal's author guidelines page. Sancy Leachman, Heidi Jacobe, Alan C. Geller, Elizabeth Stoos, Autumn Shafter, Amy Mason, Deb Girard, Theresa Malcolm, and Stephanie Savory contributed to conception and design of the study. Elizabeth Stoos, Elizabeth Bailey, Jade N. Young, Hannah Zhao, Jordan Gillespie, Hailey Pfeifer, Claudia Less, Moira Shea, Mallory deCampos‐Stairiker, Jake Smith, Alyssa Becker, Gina N. Bash, Vikram Sahni, Yichen Fan, Elena Paz Munoz, David Baron, Nadia Popovici, Victoria E. Orfaly, Wenelia Baghoomian, Emilie Foltz, Kristen Kahlen, Olivia Haddadin, Emile Latour, Kyra Diehl, Jacob Nelson, Sancy Leachman, Heidi Jacobe, and Stephanie Savory contributed to the acquisition and analysis of the data. Kyra Diehl, Jacob Nelson, Sancy Leachman, Emile Latour, and Elizabeth Stoos wrote the initial manuscript. All authors contributed to manuscript revision, read, and approved the submitted version.

## FUNDING INFORMATION

Funding for this project was from a War On Skin Cancer philanthropic fund from the Oregon Health & Science University Department of Dermatology.

## CONFLICT OF INTEREST STATEMENT

None of the authors have any conflicts of interest to disclose. This manuscript is not simultaneously being submitted elsewhere, and no portion of the data has or will be published in proceedings or transactions of meetings or symposium volumes.

## ETHICS STATEMENT

This study protocol was reviewed and approved by the institutional review board at Oregon Health and Science University (Reference STUDY00019372) on February 7, 2019. All information collected was deidentified, and IRB oversight deemed this study to pose no more than minimal risk to participants, thus no formal written informed consent was obtained.

## Data Availability

All data generated or analyzed during this study are included in this article and its supplementary material files. Further enquiries can be directed to the corresponding author.

## APPENDIX A: PRETRAINING SURVEY QUESTIONNAIRE A

### A.1

Section 1

Full Name: _______________________________________________________________________________________________________________.

E‐mail Address (If possible, please enter a work on personal email that is unique to you rather than a generic salon email.): ______________.

Industry/Profession:

() Aesthetician

() Athletic Trainer/Instructor

() Barber

() Electrologist

() Hair Stylist

() Massage Therapist

() Nail Technician

() Tattoo Artist

() Other

If you chose “Other,” please describe: ________________________________________________________________________________________.

Salon/Business Name: _____________________________________________________________________________________________________.

Address: __________________________________________________________________________________________________________________.

Unit/Suite: _______________________________________________________________________________________________________________.

City: _____________________________________________________________________________________________________________________.

State: ____________________________________________________________________________________________________________________.

Zip Code: ________________________________________________________________________________________________________________.

Phone Number: ___________________________________________________________________________________________________________.

What is your birth year? ____________________________________________________________________________________________________.

How many years have you worked in the industry? Choices 1 through 25 or more.

What is your role in your salon/business?

() Owner

() Manager

() Employee

() Other

If other, please describe: ___________________________________________________________________________________________________.

Section 2

For the next four questions, please answer based on what you see in the images provided.

For all of these questions you will see a photo of an area on a customer's skin. Some questions also include a close‐up of that same area.

Even if you are not sure, please make your best guess at the correct answer for each question.

A regular customer comes in, and you notice the spot pictured below on his skin. He notices that it has been increasing in size. How concerned are you that this should be seen by a doctor or nurse?

() Not concerned at all

() Slightly concerned

() Somewhat concerned

() Extremely concerned

While working on your client, you notice this spot on his skin. How concerned are you that this spot should be checked by a doctor or nurse?

() Not concerned at all

() Slightly concerned

() Somewhat concerned

() Extremely concerned

At a different appointment, you notice this spot pictured below on a customer's skin. She notes that the spot has not changed for several years. How concerned are you that this should be seen by a doctor or nurse?

() Not concerned at all

() Slightly concerned

() Somewhat concerned

() Extremely concerned

You saw this lesion on your customer's skin. He says it has gotten bigger over the past several months. How concerned are you that this should be seen by a doctor or nurse?

() Not concerned at all

() Slightly concerned

() Somewhat concerned

() Extremely concerned

Answer Key:

Extremely concerned. 2) Extremely concerned. 3) Slightly concerned. 4) Extremely concerned

Section 3

Over the past month, for what percentage of customers have you looked at these locations on your customers' skin for suspicious moles or lesions?

Face

() Less than 25%.

() 25–50%.

() 51–75%.

() More than 75%.

() Not applicable

Scalp

() Less than 25%.

() 25–50%.

() 51–75%.

() More than 75%.

() Not applicable

Neck

() Less than 25%.

() 25–50%.

() 51–75%.

() More than 75%.

() Not applicable

Back

() Less than 25%.

() 25–50%.

() 51–75%.

() More than 75%.

() Not applicable

Legs

() Less than 25%.

() 25–50%.

() 51–75%.

() More than 75%.

() Not applicable

Hands

() Less than 25%.

() 25–50%.

() 51–75%.

() More than 75%.

() Not applicable

Feet

() Less than 25%.

() 25–50%.

() 51–75%.

() More than 75%.

() Not applicable

Stomach

() Less than 25%.

() 25–50%.

() 51–75%.

() More than 75%.

() Not applicable.

Over the past 3 months, how many customers have asked you to look at a spot on their skin?

() None.

() 1–4.

() 5–8.

() 9 or more.

Over the past 3 months, how many times did you recommend that a customer see a doctor or nurse after you saw an abnormal mole or suspicious lesion?

() None.

() 1–4.

() 5–8.

() 9 or more times.

Over the past month, what percentage of your customers did you talk to about skin cancer during their appointment?

() Less than 25%.

() 25–50%.

() 51–75%.

() 76–100%.

How comfortable are you sharing health‐related knowledge with your customers?

() Not at all.

() A little.

() Somewhat.

() Quite.

() Extremely.

How often do you currently discuss health‐related topics with your customers?

() Never.

() Rarely.

() Sometimes.

() Often.

() Always.

Do you currently see customers in the salon/business?

() Yes.

() No.

() Not Applicable.

Before today, have you ever attended a course that included information on skin cancer?

() Yes.

() No.

() Don't know.

What is your gender?

() Male.

() Female.

() Other: _________________________________________________________________________________________________________________.

Please check one or more categories that describe you.

() African American/Black.

() Asian or Asian American.

() American Indian, Alaska Native, or Indigenous People.

() Caucasian/White.

() Hispanic, Latino, or Spanish origin (e.g., Cuban, Mexican, Mexican‐American, Puerto Rican, etc.)

() Native Hawaiian or other Pacific Islander.

() Prefer not to answer.

() Other: _________________________________________________________________________________________________________________.

## APPENDIX B Posttraining Survey Questionnaire

### B.1

Section 1

Full Name: _______________________________________________________________________________________________________________.

E‐mail Address (If possible, please enter a work on personal email that is unique to you rather than a generic salon email.): ______________.

Industry/Profession:

() Aesthetician/Esthetician

() Athletic Trainer/Instructor

() Barber

() Electrologist

() Hair Stylist

() Massage Therapist

() Nail Technician

() Tattoo Artist

() Other

If you chose “Other,” please describe: ________________________________________________________________________________________.

Salon/Business Name: _____________________________________________________________________________________________________.

Address: __________________________________________________________________________________________________________________.

Unit/Suite: _______________________________________________________________________________________________________________.

State: ____________________________________________________________________________________________________________________.

City: ____________________________________________________________________________________________________________________.

Zip Code: ________________________________________________________________________________________________________________.

Phone Number: ___________________________________________________________________________________________________________.

What is your role in your salon/business?

() Owner

() Manager

() Employee

() Student

() Other

If other, please describe: ___________________________________________________________________________________________________.

Section 2

For the next four questions, please answer based on what you see in the images provided.

For all of these questions you will see a photo of an area on a customer's skin. Some questions also include a close‐up of that same area.

Even if you are not sure, please make your best guess at the correct answer for each question.

At a recent appointment, you notice the mole pictured below on a customer's skin. She has not noticed any recent changes to the mole. How concerned are you that this mole should be seen by a doctor or nurse?

() Not concerned at all.

() A little concerned

() Somewhat concerned

() Extremely concerned

You notice the mole pictured below on a different customer's skin. She has never previously noticed it. How concerned are you that this mole should be seen by a doctor or nurse?

() Not concerned at all.

() Slightly concerned

() Somewhat concerned

() Extremely concerned

On another day, you see the spot pictured below on your customer's skin. He had never noticed the mole before. How concerned are you that this mole should be seen by a doctor or nurse?

() Not concerned at all.

() Slightly concerned

() Somewhat concerned

() Extremely concerned

A regular customer comes in, and you notice the spot pictured below. He reports that the spot occasionally bleeds. How concerned are you that this spot should be seen by a doctor or nurse?

() Not concerned at all.

() Slightly concerned

() Somewhat concerned

() Extremely concerned

Answer Key:

Extremely concerned. 2) Slightly concerned. 3) Extremely concerned. 4) Extremely concerned

Section 3

Please rate the Skinny on Skin Training you just completed.

() Not at all helpful.

() Slightly helpful

() Moderately helpful

() Very helpful

() Extremely helpful

Please tell us one new thing you learned. _____________________________________________________________________________________

The next time you observe an abnormal spot on a client's skin, how comfortable would you feel suggesting that they have it checked by a doctor?

() Not at all comfortable.

() Slightly comfortable

() Moderately comfortable

() Very comfortable

Please share your suggestions for improving the Skinny on Skin training program, or any other comments you may have. ________________

## APPENDIX C Follow‐up survey questionnaire

### C.1

Section 1

Full Name: _______________________________________________.

E‐mail Address (If possible, please enter the email you used in the original survey): _________________________________________________

Industry/Profession:

() Aesthetician

() Athletic Trainer/Instructor

() Barber

() Electrologist

() Hair Stylist

() Massage Therapist

() Nail Technician

() Tattoo Artist

() Other

State: ___________________________________________________________________________________________________________________.

Zip Code: ________________________________________________________________________________________________________________.

Phone Number: ___________________________________________________________________________________________________________.

What is your gender?

() Male

() Female

() Non‐Binary

() Prefer not to answer

Please check one of more categories that describe you:

() African American/Black

() Asian or Asian American

() American Indian, Alaska Native, or Indigenous People

() Caucasian/White

() Hispanic, Latino, or Spanish origin (e.g., Cuban, Mexican, Mexican‐American, Puerto Rican, etc.)

() Native Hawaiian or other Pacific Islander

() Prefer not to answer

() Other: __________________________________________________________________________________________________________________

Section 2

For the next four questions, please answer based on what you see in the images provided.

For all of these questions you will see a photo of an area on a customer's skin. Some questions also include a close‐up of that same area.

Even if you are not sure, please make your best guess at the correct answer for each question.

A regular customer comes in, and you notice the spot pictured below on his skin. He notices that it has been increasing in size. How concerned are you that this should be seen by a doctor or nurse?

() Not concerned at all.

() Slightly concerned.

() Somewhat concerned.

() Extremely concerned.

At a different appointment, you notice this spot pictured below on a customer's skin. He notes that the spot has not changed for several years. How concerned are you that this should be seen by a doctor or nurse?

() Not concerned at all.

() Slightly concerned.

() Somewhat concerned.

() Extremely concerned.

While working on your client, you notice this spot on her skin. How concerned are you that this spot should be checked by a doctor or nurse?

() Not concerned at all.

() Slightly concerned.

() Somewhat concerned.

() Extremely concerned.

You saw this lesion on your customer's skin. How concerned are you that this should be seen by a doctor or nurse?

() Not concerned at all.

() Slightly concerned.

() Somewhat concerned.

() Extremely concerned.

Answer Key:

Extremely concerned. 2) Slightly concerned. 3) Extremely concerned. 4) Extremely concerned

Section 3

Since taking the Skinny on Skin training course…

How many customers have you looked at a spot on their skin?

() None.

() 1–2.

() 3–5.

() 6–10.

() 11–20.

() More than 20.

How many times have you recommended that a client see a healthcare provider after you saw a suspicious mole or spot?

() None.

() 1–2.

() 3–5.

() 6–10.

() 11–20.

() More than 20.

How many of those clients returned and reported that a biopsy by a healthcare provider revealed a skin cancer?

() None.

() 1–2.

() 3–5.

() 6–10.

() 11–20.

() More than 20.

How many of the following skin cancers did your clients report, that were detected by you and later confirmed by a healthcare provider? (Example: melanoma >1, and basal cell carcinoma >2)

Melanoma: ________________________________________________________________________________________________________________

Basal cell carcinoma: _______________________________________________________________________________________________________

Squamous cell carcinoma: ___________________________________________________________________________________________________

Pre‐cancerous lesions: ______________________________________________________________________________________________________

Unknown type: ____________________________________________________________________________________________________________

How comfortable are you sharing skin cancer knowledge with your customers?

() Not at all.

() A little.

() Somewhat.

() Quite.

() Extremely.

How often do you discuss skin cancer with your customers?

() Never.

() Rarely.

() Sometimes.

() Often.

() Always

Since taking the Skinny On Skin training course, have you attended another course that included information on skin cancer?

() Yes.

() No

How often do you carefully look at your own moles, including those on your back?

() Daily.

() Weekly.

() At least once a month.

() At least every 3 months.

() At least every 6 months.

() At least once a year.

() Never

Have you had the opportunity to use a Sklip device (smartphone dermatoscope) to help a client look at their skin?

() Yes.

() No.
